# NutrimiRAging: Micromanaging Nutrient Sensing Pathways through Nutrition to Promote Healthy Aging

**DOI:** 10.3390/ijms18050915

**Published:** 2017-04-26

**Authors:** Víctor Micó, Laura Berninches, Javier Tapia, Lidia Daimiel

**Affiliations:** 1 Nutritional Genomics of Cardiovascular Disease and Obesity Fundation IMDEA Food, CEI UAM + CSIC, 28049 Madrid, Spain; victor.mico@imdea.org (V.M.); laura.berninches@imdea.org (L.B.); jtapiabelloso@gmail.com (J.T.); 2 Department of Nutrition and Bromatology, CEU San Pablo University, Boadilla del Monte, 28668 Madrid, Spain

**Keywords:** lifespan, healthy aging, caloric restriction, dietary restriction, intermittent fasting, Mediterranean diet, cardiovascular disease, type 2 diabetes, microRNAs, circulating microRNAs

## Abstract

Current sociodemographic predictions point to a demographic shift in developed and developing countries that will result in an unprecedented increase of the elderly population. This will be accompanied by an increase in age-related conditions that will strongly impair human health and quality of life. For this reason, aging is a major concern worldwide. Healthy aging depends on a combination of individual genetic factors and external environmental factors. Diet has been proved to be a powerful tool to modulate aging and caloric restriction has emerged as a valuable intervention in this regard. However, many questions about how a controlled caloric restriction intervention affects aging-related processes are still unanswered. Nutrient sensing pathways become deregulated with age and lose effectiveness with age. These pathways are a link between diet and aging. Thus, fully understanding this link is a mandatory step before bringing caloric restriction into practice. MicroRNAs have emerged as important regulators of cellular functions and can be modified by diet. Some microRNAs target genes encoding proteins and enzymes belonging to the nutrient sensing pathways and, therefore, may play key roles in the modulation of the aging process. In this review, we aimed to show the relationship between diet, nutrient sensing pathways and microRNAs in the context of aging.

## 1. Introduction

Europe is called the “old continent”, but not only for its long history. It is literally getting older. Currently, 18.9% of the European population is older than 65 years. By 2080, this proportion is expected to increase up to 28.7% [1]. Not only Europe is under this demographic pressure. Generally, developed and developing countries are facing the aging of their populations. This aging has a significant impact on demography, economy and health. For that reason, the issue of healthy aging is a global challenge. Aging is not reversible, but it is modifiable. In this regard, research should focus not only in increasing lifespan but also in improving aging quality. In the last few years, the concept of “healthy aging” has been coined to refer to disease-free aging. The aging process in developed countries is actually far from that “healthy aging” and is linked to the early development of chronic degenerative diseases, leading to chronic pharmacological treatments that start in adulthood. This is the so-called “aging phenotype.” Among the chronical-degenerative diseases associated with aging are Alzheimer’s disease (AD), Parkinson’s disease, type 2 diabetes mellitus (T2DM), cancer, cardiovascular disease (CVD), and their associated comorbidities [2]. The aim of studies focused on healthy aging is to increase disease-free lifespan. In other words, not only to live longer but also to live better.

The first step on this scientific road is to gain full knowledge about the biological aspects of the aging process. A recent workshop addressed this questions with the aim of identifying essential aging pathways and reaching a consensus about most promising approaches (pharmacological, dietary and behavioral) to extend longevity and delay the onset of age-related diseases [3]. In this regard, some biological processes have been described to be altered by age, including genetic and epigenetic factors, telomere shortening, proteostasis, signaling pathways impairment, reduction of stem cell pluripotency, cellular senescence, mitochondrial dysfunction, immune dysfunction and alterations in nutrient sensing pathways [4]. Alterations in nutrient sensing pathways have gained increasing attention in the field of aging because they can be modulated both pharmacologically and with dietary interventions [3]. Some of the nutrient sensing pathways impaired by aging are the IGF1/PI3K/AKT/mTOR and AMPK/Sirtuin/PGC1α pathways. These pathways play key roles in the regulation of protein synthesis, cell cycle, DNA replication, autophagy, stress response and glucose homeostasis.

Secondly, we must identify the environmental factors that modulate the biological pathways involved in aging. Among the environmental factors modulating the aging process are smoking, pollution, physical activity and diet [5]. Diet is one of the environmental factors that has the greatest impact on the aging process and contributes to the quality of such a process. In this regard, it has been suggested that caloric restriction (CR) is a successful dietary approach to increase lifespan in a healthy manner [3,6]. Diet and dietary compounds may well affect those molecular aging pathways mentioned above and, thus, modulate the healthiness of the aging process.

MicroRNAs are short non-coding regions of 22–25 nucleotides that regulate a plethora of cellular processes. They bind to the 3′-UTR sequence of their target genes and reduce their expression by promoting mRNA degradation or impairing protein translation. MicroRNAs can also be found in the circulation and these circulating microRNAs have been proposed to be important biomarkers of disease status and progression [7]. Moreover, microRNAs can be modulated by diet and can even been obtained from dietary resources [8]. Recent studies have shown that microRNAs can also modulate age-related processes, including DNA replication, cellular senescence and apoptosis [9]. MicroRNAs have also been shown to modulate nutrient sensing pathways [10,11]. We propose the use of the term “miRaging” to refer to those microRNAs with a role in the aging process or that have been associated with longevity in model organisms as well as in humans. Therefore, microRNAs modulate nutrient sensing pathways and can be modulated by diet. This review aims to describe the intricate network that links diet, healthy aging and miRagings.

## 2. Dietary Interventions to Increase Lifespan and Promote Healthy Aging

Several studies have suggested that diet can slow aging and, consequently, increase healthy lifespan. Studies in model organisms such as *Caenorhabditis elegans*, mice and non-human primates point to a CR-based intervention as a promising tool to fight against aging [12]. However, human studies, specially intervention trials, are scarce and there is a current controversy about what kind of CR-based therapy is safe and effective in extending human healthy life expectancy.

CR has been shown to extend lifespan in different model organisms. In the early 80s, Yu et al. showed that at least 60% of the specific pathogen free Fischer 344 rats in the CR group lived longer than rats fed ad libitum [13]. CR rats also showed less age-related lesions at death [13]. Moreover, middle CR attenuated the age-related decline in autophagy and age-related increase in oxidative stress in rat skeletal muscle [14] and showed a lower decline in insulin sensitivity in rat liver [15]. Mice submitted to an intermittent fasting (IF) dietary restriction regime also showed better regulation of glucose homeostasis [16] that could be mediated by a 40% reduction in the IGF-1 levels, although a hyperphagic response to fasting was observed [17]. However, in a later study, mice submitted to a 100% CR did not show higher food intake on the feast day [18]. A recent meta-analysis concluded that there is enough evidence pointing to the CR-mediated increase in lifespan in rodent models, although there is an important genetic effect on mice strains that result in a great variety of responses to dietary interventions [19]. A complete removal of food has been shown to extend lifespan of *Caenorhabditis elegans* worms [20,21]. In non-human primates, the Wisconsin National Primate Research Center (WNPRC) did show improved survival associated with 30% CR in adult rhesus monkeys with less incidence of age-related diseases and less loss of grey matter [22]. On the contrary, the National Institute of Aging (NIA) study on rhesus monkeys indicated that CR did not extend lifespan irrespective of whether the CR was started in young or old monkeys [23]. Several differences in the experimental design could explain these contradictory results [12,24], and highlights the need for further validation of current studies in different animal models.

Some human observational studies suggest that CR could be also effective in extending human lifespan. It is well-known that the number of centenarians in Okinawa, a Japanese island, is surprisingly high. This extremely high local life expectancymay well be due to the fact that Okinawa inhabitants traditionally follow a diet that is low in proteins and rich in vegetables, fruits and fish and that the intake of Okinawans is a 17% lower than the Japanese average intake, in addition to Okinawans having a higher isoflavone intake [25]. Interestingly, the Okinawan diet is rich in monounsaturated and polyunsaturated fatty acids [2], a characteristic that is shared by the Mediterranean Diet. Mediterranean Diet has also been postulated as a powerful nutritional tool to promote healthy aging [26], especially because it reduces CVD mortality [27]. Results from the PREDIMED interventional trial have shown that a greater adherence to the Mediterranean Diet is associated with longer telomeres [28]. Moreover, longer telomeres at baseline was associated with a better response to the dietary intervention [29]. Higher intake of n-3 polyunsaturated fatty acids has been associated with better cognitive performance and lower cognitive decline in both the Mediterranean and the Okinawan diets [30,31].

Especially interesting are the human interventional trials that directly address the effect of CR on human health and longevity. The Pennington CALERIE Team carried out a randomized trial with healthy sedentary men and women based in six months of a medium CR (25%), intensive CR or a CR with physical activity (PA) (12.5% CR + 12.5% increase in energy expenditure through exercise) intervention. They showed that body temperature, glucose and insulin levels, well-known longevity biomarkers, decreased with CR with or without PA. However, there was no beneficial effects of the intensive CR intervention [32]. The two-year CR intervention carried out as part of the CALERIE 2 study did not find a significant decrease in core temperature in the CR group compared with the ad libitum group. However, the lower adherence to the CR protocol found in this study compared to the CALERIE 1 study may have influenced the results observed about the effect of CR on body temperature and other age-related biomarkers [33]. Further studies of the Pennington CALERIE Team have shown that CR reduces liver lipid content [34] and improves CVD risk [35]. On the contrary, this intervention increases fasting ghrelin levels (the so-called “hunger hormone”) and does not modify growth hormone [36] nor cortisol levels [37]. Interestingly, a 6–12 months long CR intervention resulted in metabolic and behavioral adaptations that led to an improvement in physical functioning and vitality [38] without inducing eating disorder symptoms [39]. Therefore, results from the CALERIE study enormously contribute to the knowledge about how CR affects human health and point to the beneficial effects of CR on longevity biomarkers. However, some results need to be further validated to finally elucidate the effects of CR, especially when sustained for long periods of time on human longevity. An insight into the long-term effect of severe CR comes from the observational CRON study. The CRON study takes advantage of the data provided by members of the Calorie Restriction Society that have been following an approximately 30% CR intervention for 15 years. They showed lower total and LDL-cholesterol levels, lower triglycerides, blood pressure, fasting glucose and fasting insulin levels and higher HDL-cholesterol levels than age-matched controls eating a usual Western diet [40]. Other age-related biomarkers such as core body temperature and inflammatory interleukins were also reduced, strongly suggesting that this kind of sustained CR can effectively extend lifespan [41,42]. In this regard, a longer follow-up of this population is warranted in order to determine whether this intervention will finally result in longer lifespan.

An alternative to a sustained CR diet is an alternate fasting therapy studied in the FEAST trial. Results from this study showed that this intervention led to a 4% weight reduction, a decrease in fasting insulin levels and an increase in fatty acid oxidation, although fasting glucose levels were not affected [43]. These results were further confirmed when authors found that alternate fasting resulted in an increase in the expression of genes involved in fatty acid oxidation, but that there was no a beneficial effect on glucose homeostasis [44]. However, another study showed that alternate CR can be effective in reducing insulin resistance in severe insulin-resistant individuals [45]. The FEAST study also showed that this is a suitable approach in humans, as hunger in fasting days did not increase [43]. Another study showed that alternate-day CR did not counteract the negative effects of eight days of bedrest regarding mood, insulin resistance and visceral fat accumulation [46]. In young obese/overweight women, it has been shown that both IF and CR are equally effective for reducing weight, leptin levels, total cholesterol, LDL-cholesterol and triglycerides and increasing insulin sensitivity [47].

Overall, these studies conclude that CR has a positive effect on human health and, thus, can greatly contribute to extending lifespan and promoting healthy aging. This allegation is shown in the recent meta-analysis carried out by Lettieri-Barbato et al. that included 43 studies, both experimental and observational [48]. They showed that CR decreases total and visceral fat accumulation, levels of inflammatory biomarkers as well as levels of leptin, and that it increases adiponectin levels [48]. However, CR also leads to metabolic adaptations that include a decrease in energy expenditure and loss of muscle mass and strength [6,49] that can be detrimental if CR is sustained in the long run. Others studies assert that the effect of CR on longevity is actually due to the concomitant restriction of proteins or specific amino acids [3]. In yeast, the restriction of the amino acids serine, threonine and valine promote stress resistance and longevity [50]. The study carried out by Mair et al. showed that withdrawing the yeast extract, that is, the protein source, of the *Drosophila melanoganster* had a much greater effect on lifespan extension than glucose restriction, and was independent of the caloric intake [51]. Tryptophan restriction has been shown to be related to longevity in rats [52,53]. Finally, methionine restriction has been also showed to impact lifespan. For instance, reduction of l-methionine from 0.86% to 0.17% of the diet increased the lifespan of male Fisher 344 rats by 30% [54]. A methionine-deficient diet also increased the lifespan in a mouse model. Mice in this diet showed lower levels of IGF-1, insulin and glucose and a higher resistance to liver injury promoted by induced oxidative stress [55]. The amino acid sensors GCN2 and mTOR are suggested to be involved in the beneficial effects of protein or selective amino acid restriction in longevity [56].

In summary, the evidences pointing to a positive effect of CR on longevity are promising. However, some aspects need to be further studied and, in this regard, an extensive debate is taken for granted. Indeed, to bring CR into clinical practice some limitations must be overcome: (1) the definition of a standardized panel of biomarkers of the effect of CR on aging and (2) the standardization of the CR intervention. Regarding issue number 2, different approaches are still under examination to determine their feasibility and safety; chronic CR vs. IF, time-limited food intake vs. standard 3–5 meals per day diets, and total intake CR vs. specific amino acids CR. It is also necessary to define the most suitable age of intervention onset as well as the genetic polymorphisms that can modulate the individual response to a CR intervention.

## 3. Nutrient Sensing Pathways: Their Role in Molecular Aging

**Figure 1:**
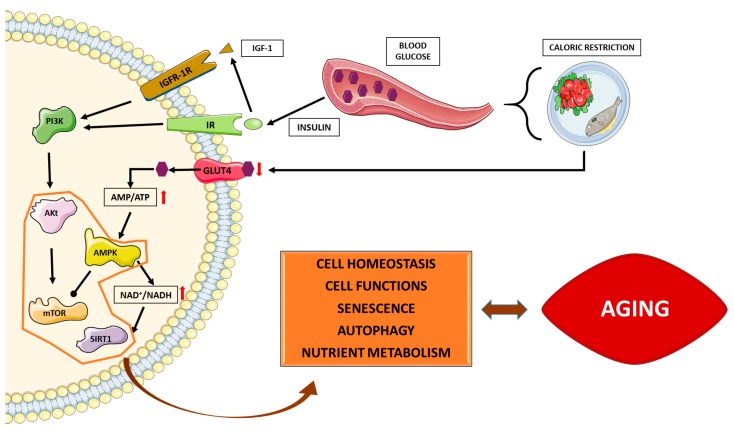
Caloric restriction (CR)-mediated modulation of nutrient sensing pathways. CR results in a decrease in plasma glucose levels that, in turn, decrease IGF-1 and insulin levels. As a result, the signaling downstream insulin receptor (IR) and IGF-1R decreases. In this situation, AKT is in its un-phosphorylated inactive form and, thus, mTOR is also inactive. The decrease in the cellular availability of glucose also increases AMP/ATP ratio and, consequently, AMPK is activated. AMPK also inhibits mTOR complex 1 and increases NAD^+^/NADH ratio. SIRT1 is a histone deacetylase activated by NAD^+^. Coordinately, these interconnected nutrient-sensing pathways modulate cell homeostasis, cellular function, senescence, autophagy and metabolism, contributing to healthy aging and longer lifespan. Images from Servier Medical Art have been included in this figure (Available online: http://www.servier.com/Powerpoint-image-bank).

The mechanisms by which CR promote longevity are still not completely understood. However, some animal and human studies suggest that insulin/IGF-1/PI3K/AKT, mTOR and AMPK/SIRT1 pathways are involved [3,57,58,59]. These pathways link metabolism, diet and aging. High glucose levels induce insulin release that, in turn, increases IGF-1. IGF-1 binds to its receptor, switching on its autophosphorylation, and leading to the subsequent activation of PI3K. PI3K phosphorylates and activates AKT. Activated AKT phosphorylates and activates mTOR and inhibits FOXO. CR-mediated reduction of glucose levels affect this signaling cascade (Figure 1).

It has been shown that a decrease in mTOR signaling leads to a lifespan extension in yeast, worms, flies and mice [57]. Mice treated with rapamycin, a pharmacological mTOR specific inhibitor, lived longer, although the effect seemed to be higher in female than in male mice [60]. The mechanism by which pharmacological inhibition of mTOR signaling increases longevity is not clear, since rapamycin did not mimic the effect of dietary restriction on insulin, IGF-1 or leptin levels and even worsened glucose tolerance [61]. FOXO is a broadly conserved subfamily of transcription factors that are involved in key longevity pathways such as stress response, antioxidant activity, cellular proliferation, apoptosis and autophagy [59]. Moreover, FOXO transcription factors can contribute to extreme longevity in humans and model organisms. In *Caenorhabditis elegans*, it has been shown that a decrease in insulin/IGF-1-like signaling promotes stress resistance and longevity through the inhibition of FOXO protein DAF-16 and the nuclear accumulation of SKN-1. The latter was mediated by AKT1/2 and SGK-1 phosphorylation independently of DAF-16 inhibition [62]. A regimen of IF based in fed/fasting cycles of two days confirmed the role of DAF-16 in worm lifespan extension and, additionally, identified AP-1 transcription factor as a modulator of IF-induced longevity [63]. Although dFOXO, the equivalent of mammalian FOXO3, is not necessary for the dietary restriction-mediated increase in lifespan, it contributes to the modulation of the effect of the dietary restriction in flies [64]. In mice, the effect of dietary restriction on lifespan extension seems to need FOXO3 [65]. The role that FOXO3 plays in oxidative stress or autophagy could mediate its effect on dietary restriction-mediated lifespan extension [59,66]. In humans and rats, CR resulted in transcriptional and post-transcriptional modifications of genes belonging to the PI3K/AKT/FOXO pathway. Interestingly, some polymorphisms in *FOXO3* gene have been associated with longer lifespan [67] and with lower total and coronary heart disease mortality [68].

In humans and rats, CR increased pathways such as muscle contraction, glycolysis and gluconeogenesis, oxidative phosphorylation and mitochondrial function, whereas other pathways like insulin/IGF-1 signaling and “aging brain” were downregulated [69]. Seventy-two hours of fasting in CD-1 mice reduced plasma IGF-1 levels up to 70%, whereas the levels of IGF-1 binding protein, which reduces IGF-1 signaling, were increased 11.4 fold [70]. In flies, dietary restriction also increased lifespan through a decreased TOR activity [71]. In *Caenorhabditis elegans*, the mutation of the equivalent to mTOR or a component of mTORC1, *raptor*, could extend lifespan [72]. An IF regime in *Caenorhabditis elegans* has been shown to increase the worm lifespan by a mechanism that involves RHEB-1, an mTORC1 activator [73]. However, another experiment based in the complete food deprivation of the worms showed that the extension of lifespan was independent of the insulin/IGF-1 signaling pathway and the sirtuin Sir-2 [20,21]. Also, studies in different organisms have shown that rapamycin, an immunosuppressor drug that targets mTORC1, increases lifespan [57]. Which kind of dietary intervention modulates mTOR is currently under debate, because it seems that mTOR is more responsive to a protein-restricted diet than to complete CR diets [12]. In a xenograft mouse model of prostate and breast cancer, protein restriction decreased tumor growth and intra-tumor mTOR activity. Interestingly, a low-protein diet also decreased serum IGF-1 levels [74]. Low-protein/high-carbohydrate diets increased mice longevity, probably through a decrease in mTOR activation [75]. In humans, long-term CR did not decrease serum IGF-1 levels. However, individuals who were usually following a low-protein dietary regime showed lower IGF-1 levels [76]. Moreover, polymorphisms in the gene encoding the IGF-1 receptor that impairs IGF-1 signaling and results in higher plasma concentration of IGF-1 have been associated with extreme longevity [77]. Lower plasma IGF-1 levels have also been associated with longer survival in nonagenarian women, but not in males [78].

Serum collected from individuals of the FEAST study showed reduced proliferation of HepG2 cells and increased SIRT1 expression [79]. This increase in SIRT1 levels has also been found in cells incubated with the serum of CR participants of the CALERIE study [79] and in muscle samples of the participants of the FEAST study [44]. SIRT1 is a NAD^+^-dependent deacetylase with a key function in energetic metabolism, due to its role as an NAD^+^ sensor [80]. Studies in yeast suggest that NAD^+^ synthesis and Sir2 activation are needed for lifespan extension [81]. In a mouse model of Cockayne Syndrome, an accelerated aging disorder, it has been shown that premature aging results from an impairment in the DNA repair system that leads to a decrease in SIRT1 activity [82]. Moreover, SIRT1 protein levels increased in specific brain areas of mice under a dietary restriction regime [83] and mice overexpressing SIRT1 in the brain showed significant lifespan extension [84]. The mechanism by which SIRT1 contributes to lifespan extension is still unclear. However, it has been shown that SIRT1 increases the expression of some genes involved in neuronal signaling and SIRT1 overexpressing mice showed enhanced neuronal activation and an improved neuronal adaptation to dietary restriction [83,84]. These studies highlight the potential usefulness of strategies focused in SIRT1 modulation to promote healthy aging. The use of synthetic activators of sirtuins is currently under debate due to several safety concerns. For that reason, before these activators could be implemented, the precise role of SIRT1 in lifespan extension needs to be further clarified [85]. Interestingly, some phytochemicals like resveratrol are able to activate SIRT1 in vitro [86]. Like mTOR, AMPK is a cellular energetic sensor activated by a high AMP/ATP ratio, that is, when cellular energy is low. It is well known that AMPK activation has an insulin sensitizer effect. This suggests that AMPK could play a role in aging. Actually, metformin treatment has been proved to extend lifespan in worms, rats and mice [87]. Metformin is a hepatic AMPK activator. In humans, metformin treatment reduced the onset and progression of age-related diseases such as CVD, cancer and cognitive decline [3,88,89].

Studies carried out in the last decade have highlighted the role of gut microbiota on animal health. Some recent studies also showed that gut microbiota play a key role in longevity. In this regard, some studies have shown that feeding worms with different bacterial species instead of the standard laboratory *E. coli* OP50 strain significantly affects lifespan [12]. Dietary restriction leads to alterations in the composition of the gut microbiota and such alterations may be important contributors to the effect of dietary restriction on longevity. For instance, the study of Zhang et al. showed that CR had a stronger effect on gut microbiota architecture than exercise [90]. They showed that the mice submitted to a CR low-fat diet had a different gut microbiota profile than the others. Also, the age-related changes in the gut microbiota composition of the mice were different in CR groups [90]. The small intestine holds nutrient-sensing mechanisms that play a key role in glucose homeostasis [91]. This nutrient-sensing mechanism involves protein kinase C, glucokinase and glucokinase receptor 1 that, collectively, form the PKC-CCK-CCK1R pathway activated by long-chain fatty acids in the duodenum. This activated pathway triggers a signal through the vagal nerve to the central nervous system that, in turn, sends the order to lower glucose production in the liver [91]. Fatty acids also trigger a nutrient-sensing response through a family of G-protein coupled receptors named GPR40 and GPR120 that, upon activation, lead to an increase in pancreatic insulin secretion and trigger PI3K/AKT signaling in adipocytes [92]. The infusion of intestinal microbiota from lean donors into the duodenal tube of obese individuals leads to an improvement in insulin sensitivity, although the mechanism involved in this effect is still unknown. [93]. It has been hypothesized that the mechanism by which gut microbiota modulates CR-mediated increase in lifespan is through the modulation of gut nutrient-sensing mechanisms [91]. However, this hypothesis need to be experimentally addressed.

Collectively, these studies suggest that the modulation of these nutrient sensing pathways is fundamental to modulating the aging process. However, most of these results come from animal studies, thus more human studies are needed to fully understand the role of these pathways in human aging and to clarify how they can be effectively targeted, nutritionally or pharmacologically, to safety prolong life.

## 4. MiRagings: What and Which Are They? How Can They Be Modulated by Diet?

Non-coding RNA sequences are some of the molecular players of aging [94]. These sequences are some of the most important mechanisms of epigenetic regulation of gene expression [95]. The most representative of these sequences are microRNAs, non-coding RNA sequences of ≈22 nucleotides that are located in intra- or inter-regions of protein coding genes. Their main function is the inhibition of their target genes, and they are involved in the regulation of many cellular processes such as cellular proliferation, apoptosis and cellular metabolism, among others [96]. In addition to this, microRNAs are present in plasma and other body fluids such as urine and cerebrospinal fluid [7]. Circulating microRNAs are usually associated with exosomes, lipoproteins and protein complexes due to the necessity of protection from RNAses degradation. Circulating microRNAs are in the spotlight due to their potential value as biomarkers of health, disease and nutritional status. Modification of circulating microRNA profiles are associated with cholesterol metabolism, T2DM, CVD, insulin sensitivity, endothelial function, inflammation and aging [7]. MicroRNAs synthesis has been well described. RNA polymerase II transcribes microRNAs inside the nucleus, where a hairpin pri-miRNA precursor is generated. This hairpin pri-miRNA is firstly processed by Drosha to form a pre-miRNA molecule of 70 nucleotides, approximately. This pre-miRNA is exported to the cytoplasm via Exportin 5, where the maturation process will be completed by DICER, resulting in the mature microRNA. This mature microRNA is associated with Argonaute proteins to form an RNA-induced silencing complex (RISC), which guides the mature microRNA to its target gene [97].

We can define miRagings as those microRNAs that modulate biological processes related to aging or whose expression changes with age. Given that diet has been shown to affect longevity and that nutrient sensing pathways are important mediators of that effect, we hypothesize that miRagings could be the link between diet, aging and nutrient sensing pathways. Then, which are those “miRagings”? How can they regulate nutrient sensing pathways? Also, how are they modulated by CR or other dietary compounds? By answering these questions, we can start to understand the role of microRNAs in aging and, most importantly, we can figure out how to use them as tools to reach the goal of healthy aging. Table 1 summarizes the link between each mentioned miRaging, the nutrient sensing pathways and diet.

microRNA expression could be affected by age, and some age-modulated microRNAs target genes belonging to the nutrient sensing pathways. The mechanism by which aging modifies microRNA expression is still unknown. However, it has been described that the age-related increase in stress leads to the upregulation of p53. p53, among other functions, could have an influence on the Drosha complex, thus affecting microRNA maturation [98]. In addition, the onset and progression of age-related diseases such as T2DM, CVD, inflammation, and cancer are modulated by microRNAs [99,100,101]. Regarding T2DM, it is known that the let-7 family modulates glucose homeostasis and insulin sensitivity [102,103]. Other microRNAs that regulate the insulin signaling pathway are miR-33, miR-103, miR-107 and miR-29 [99,104,105]. Circulating miR-1, miR-208a and miR-133a are overexpressed in the following 2 h after an acute myocardial infarction [106], and circulating miR-423-5p is upregulated in heart failure [107,108]. These results demonstrate the potential of circulating microRNAs in the diagnosis of CVD. Aging is linked to chronic low-grade inflammation. Some microRNAs that could be highlighted as regulators of inflammation are miR-146, miR-155 and miR-21 [109]. Moreover, miR-155 and miR-16 were found to be upregulated in B-cells of elderly subjects compared to young subjects [110].

Apart from the well-known deregulation of microRNAs in age-related diseases, miRNA profiles (specially circulating miRNAs) also change with age [9,111]. It is worth mentioning a study carried out by Serna et al. who studied circulating microRNA profiles in 36 subjects (20 centenarians and 16 octogenarians) in peripheral blood mononuclear cells (PBMCs), and found that microRNA profiles in centenarians were more similar to the profiles of young adults than those of octogenarians [111]. These results could suggest that a specific circulating microRNA profile will allow us to predict longevity. Circulating microRNA profiles could additionally allow us to discriminate between healthy and unhealthy aging and could be used to prevent disease onset before its occurrence [112]. A recent study in humans and mice demonstrated that the circulating levels of miR-34a changes with the development of age-related hearing loss [113]. Another study carried out in Wistar rats showed that microRNA expression changes during aging. Authors specifically found the upregulation of miR-34a, miR-124a and miR-383 and the downregulation of miR-130b and miR-181a. They suggested that this change could contribute to the failure of pancreatic β cells observed that results in insulin resistance [114]. Indeed, miR-124a has been associated with glucose-induced insulin secretion through the direct modulation of *AKT3* and *FOXA2* and, potentially, *SIRT1* [115]. Studies in SH-SY5Y and SK-N-SH cell lines suggest the possible effects of miR-124 on neuronal apoptosis and autophagy in Parkinson’s disease [116]. Moreover, miR-124 is overexpressed in aged skin when compared with its expression in young skin [117]. miR-130b and miR-181a regulate the PI3K/AKT pathway through the inhibition of *PTEN*, a negative regulator of this pathway [118,119]. miR-181a also directly inhibits *SIRT1*, resulting in decreased insulin sensitivity [120]. Interestingly, circulating levels of miR-130b increase after an intervention with polyunsaturated fatty acids in women [121]. Another study showed that miR-34 is upregulated in age-related macular degeneration [122]. Moreover, it has been reported that miR-383 modulates the insulin signaling pathway through IGF-1 and its receptor [123], and that it is downregulated by a high-fat diet (HFD) in mice pancreatic islets [124]. Finally, in human IDH4 fibroblasts, let-7 is related to the expression of *p66SHC*, which is implicated in cellular senescence [125].

All these studies show that aging significantly modulates the expression many microRNAs. However, the significance of this age-related microRNA modulation is currently unknown and the study of the role of microRNAs in aging is still in its infancy. The molecular pathways affected by age-related microRNAs are currently under investigation. In this sense, some recent studies point to an important influence of microRNAs on aging processes such as deregulation of nutrients sensing pathways, immune system dysfunction, cellular damage and age-associated diseases [4]. In this review, we focus on the role of microRNAs as modulators of nutrient sensing pathways related to age.

The IGF1/PI3K/AKT/MTOR pathway is regulated by let-7 expression, a microRNA that targets multiple components of the IGF1 pathway as the IGF1 receptor or mTOR [11]. In *Drosophila melanogaster*, miR-200 and miR-8 are important regulators of PI3K through targeting *USH/FOG2* [126]. Another study in myoblasts demonstrated that miR-432 is a negative regulator of myoblast proliferation and differentiation through the modulation of the PI3K/AKT/mTOR pathway [127]. In a mouse model, the regulation of this pathway has also been found to be mediated by miR-1, which is downregulated in many cancers and inhibits cancer cells growth and proliferation, and promotes apoptosis [128]. The role of miR-1 in aging has been revealed in a progeria mouse model, where it has been found that miR-1 is upregulated in liver irrespective of GH levels [129]. Another study in mice has shown that this microRNA can be modulated by diet, as it has been found to be downregulated in the adipose tissue of mice fed an HFD [130]. miR-155, which has been associated with age [109,110], enhances insulin sensitivity through the modulation of the AKT pathway [131] Moreover, miR-223, a microRNA that targets this pathway, is involved in the regulation of mast cell apoptosis in rat basophilic leukemic cells [132]. In comparison of young and old donors, miR-223 levels were decreased in CD4+ T cells [133]. In another mouse model, the deregulation of mTOR mediated by the miR-17-92 cluster has been shown to produce the disruption of Sertoli cell polarity and spermatogenesis [134]. Age-related reduction of miR-17-92 will result in more oxidative stress and DNA damage [135]. In addition to this, in murine differentiating skeletal muscle cells the overexpression of IGF1 produces a downregulation of miR-146a [136]. In colorectal cancer, re-expression of miR-145 produces the repression of *IRS1* and *IRS2* [137]. MiR-145 expression also decreases in PBMCs of aging humans [138].

The AMPK/Sirtuins/PGC1-1α pathway is also regulated by microRNAs. For instance, let-7 regulates SIRT1 expression in human biliary epithelial cells [139]. *SIRT1* is also downregulated by miR-217, leading to the modulation of endothelial cell senescence via silent information regulator 1 (SIR1) [140]. Apart from SIRT1, miR-133 also inhibits *AMPK* expression. Thus, miR-133 targets this pathway at two different points [99]. In humans, Kurylowicz et al. observed that the downregulation of *SIRT1* negatively correlated with the expression of miR-22-3p in obese individuals and the upregulation of *SIRT7* negatively correlated with miR-125a-5p levels in slim individuals [141]. Another study in humans showed the importance of the regulation of miR-199 as a modulator of SIRT1 and as a biomarker of atrial fibrillation after coronary artery bypass graft surgery [142]. In rhesus macaque bone marrow, these microRNAs showed age-related downregulation [143]. MiR-19b/221/222 are important regulators of PGC1-1α. Interestingly, miR-19b was found to be downregulated in octogenarians, while centenarians and young people conserved the same level of this microRNA [111]. miR-19b could also be a biomarker of polyunsaturated fatty acids intake, as its circulating levels increased in women after eight weeks of a normocaloric diet enriched in these fatty acids [121]. In atherosclerosis, these microRNAs could induce endothelial cell dysfunction through the downregulation of *PGC-1α* [144]. In adipose-specific miR-455 transgenic mice, it has been found that miR-455 activates AMPK in brown adipose tissue (BAT), suggesting the importance of this miRNA in BAT adipogenesis through the regulation of the AMPK/Sirtuins/PGC1-1α pathway [145]. In addition to this, miR-455 is downregulated in old mice [146] and upregulated in the liver of HFD-fed mice [147]. In HT-29 cells and HEK-293 cells lines, miR-451 produces an inhibition of AMPK and an activation of mTORC1 [148]. In aging primates, an upregulation of miR-451 in skeletal muscle associated with CR has been found [149]. In human melanoma cell lines and melanoma tissues, Liu et al. found a downregulation in miR-425. The authors suggested that miR-425 could be a tumor suppressor acting through the inhibition of the PI3K/AKT pathway [150]. In other cells lines, miRNA-221 induces apoptosis via the modulation of KIT/AKT [151]. In bone marrow-derived mononuclear cells, IGF-1 blocks the processing of miR-34a [152]. Also, in a rat model of aging brain it has been shown that the effect of miR-34a on aging could be mediated by SIRT1/mTOR pathways [153]. Finally, in vitro and in vivo models show that miR-16 inhibits cell proliferation by targeting *IGF1R* [154]. It is important to highlight the role of miR-144 as a regulator of these pathways. This microRNA regulates the IGF-1/PI3K/AKT pathway by targeting *PTEN* [155] and *IRS1* [156], but it also directly targets *MTOR* [157] and *AMPK* [158].

Unfortunately, human studies addressing the role of microRNAs on the modulation of these age-related nutrient-sensing pathways are scarce. Olivieri et al. found a downregulation of miR-182, miR-223 and miR-142-3p in the skeletal muscle of postmenopausal women [159]. These microRNAs regulate *IGF-1R* and *FOXO3A* expression, as well as activate insulin/IGF-1 pathway signaling via the phosphorylation of AKT and mTOR [159]. Interestingly, miR-142-3p has been found to be upregulated in the adipose tissue of mice fed an HFD [130], and miR-182 has also been found to be upregulated in the liver of mice fed an HFD [160]. Another study in humans showed the influence of miR-4458 in the regulation of *IGF-1R*. MiR-4458 levels are higher and inversely correlated with *IGF-1R* levels in lumbar disc degeneration patients, suggesting a role of this microRNA in the development of this condition [161]. Moreover, in humans, miR-613, which targets c-MET and PI3K/AKT/mTOR pathways, is downregulated in osteosarcoma and its downregulation is associated with lymph node metastasis [162].

Different strategies could delay aging or contribute to healthy aging. These strategies can be based in longevity drugs or lifestyle interventions. Regarding lifestyle interventions, we have described that CR has been proved to be effective in different animal models. The mechanisms by which CR extends lifespan is not fully understood, but microRNAs could play a key role. In rats, CR reduces miR-144 expression in cerebromicrovascular endothelial cells, avoiding the reduction of *NRF2*, a regulator of cellular resistance to oxidants [163]. In old rhesus monkeys, this microRNA was found to be upregulated in skeletal muscle [149], although CR dampened this upregulation [149]. In this same work, other microRNAs associated with age that regulate the PI3K/AKT pathway and SIRT1, increased in old monkeys submitted to a CR intervention [149]. Apart from these microRNAs, it must be highlighted the role of miR-221 that is downregulated by CR [149] and polyunsaturated fatty acids [121]. In murine models, a group of miRNAs (miR-425, miR-196, miR-155, miR-150, miR-351, miR-16, let-7, miR-34, and miR-138) were differentially expressed between the control and CR groups [164]. Interestingly, some microRNAs that are differentially expressed by CR, have been described as regulators of nutrient sensing pathways (let-7, miR-34, miR-425, miR-16, miR-155, miR-144, miR-451). The cluster miR-17-92, whose role in longevity and regulation of nutrient sensing pathways has been mentioned before, is also downregulated by CR in a mouse model of breast cancer [165]. On the contrary, miR-145 does not seem to be regulated by CR, although it is upregulated by an HFD [166]. It is worth mentioning that other dietary approaches to increase longevity are currently under debate, for example, protein restriction could also modify the expression levels of some miRagings. In this regard, it has been shown that a low-protein diet reduced the expression of miR-124a in pancreatic islets of pregnant rats [167], whereas a high-protein regime decreased HDL-associated miR-223 levels in overweight or obese men [168]. All these studies show that many miRagings are modulated by longevity nutritional approaches such as CR or protein restriction. However, whether this modulation results in increased longevity still needs to be further elucidated. Indeed, the effect of CR on microRNA profiles is still unclear due to the lack of studies in this field. For this reason, human studies that combine CR with microRNA profiles are needed. The potential of microRNAs as biomarkers of aging highlights the future importance of them as biomarkers of the effect of moderate CR on human longevity and healthy aging.

## 5. Conclusions and Future Perspectives

In the following decades, the population of developed and developing countries will grow older and older, resulting in a demographic shift that, consequently, will lead to a higher prevalence of degenerative diseases. For this reason, aging is a major concern worldwide and the necessity of dealing with this challenge is well recognized. The promotion of healthy aging can contribute to the amelioration of these consequences by reducing the prevalence of chronic-degenerative diseases and delaying their onset. The aging world calls for the development of reliable and effective policies to deal with the consequences of aging. In 2013, a workshop entitled “Interventions to Slow Aging in Humans: Are We Ready?” was celebrated in Erice. There, leading experts in the field of healthy aging gathered to define the most important aging mechanisms and reach a consensus about the most promising approaches to slow aging [3]. They concluded that current research supports the usefulness of dietary restriction as a tool to increase healthy lifespan. They also identified nutrient sensing pathways as essential molecular pathways that modulate aging. Thus, pharmacological targeting of these pathways could be considered as a promising approach to extend healthy lifespan. However, before all these strategies can be brought into practice, it is necessary to fully understand the molecular mechanisms that mediate the effect of dietary interventions on aging, as well as to standardize an effective and safety dietary intervention. The safety of long-term CR or fasting (both prolonged and IF) should be cautiously considered, especially in elderly people, diabetics or other non-healthy individuals.

Current research in model organisms suggests that diet has a more prominent role on aging and age-related diseases than previously hypothesized. However, the precise dietary intervention that effectively increases healthy lifespan is not defined so far. In this regard, more human intervention studies and a deeper knowledge of the molecular mechanisms and environmental factors modulating aging are needed.

Recent studies have shown that epigenetic markers can be modified by environmental factors, with diet being among them. MicroRNAs have recently emerged as important epigenetic regulators of cellular function. They can be modified by diet. Here we have reviewed how CR affects levels of some microRNAs. Interestingly, some of these CR-modulated microRNAs target genes encoding nutrient sensing proteins associated with molecular aging and are modified along the aging process. We have defined those microRNAs as “miRagings” and we hypothesize that these microRNAs that link diet and nutrient sensing pathways can be used to modulate aging. It has been suggested that the pharmacological inhibition of IGF-1 or mTOR pathways can be considered as a potential tool to slow aging [3]. MicroRNAs that modulate these pathways are also pharmacological targets for the development of new therapies to slow aging. However, whether the modulation—nutritional or pharmacological—of these miRagings results in a prolonged healthy lifespan is not currently known. In fact, the study of the role of microRNAs in aging is still in its infancy and, in this regard, more human and animal studies aimed at defining a panel of modifiable age-related microRNAs are needed. Once these microRNAs have been defined, some human trials are needed to study their safety and effectiveness in prolonging healthy life expectancy.

## Figures and Tables

**Table 1:** List of miRagings, their effect on nutrient sensing pathways and how they may be affected by diet.

MicroRNA	Relationship with Aging	Related Nutrient Sensing Pathway	Effect of Diet	References
let-7	Associated with the regulation of cellular senescence	IGF-1/PI3K/AKT mTOR SIRT1	It is differentially expressed because of CR	[11,125,139,153,164]
miR-1	Upregulated in the liver of progeria models	IGF-1/PI3K/AKT mTOR	Downregulated in the adipose tissue of mice fed a high-fat diet	[128,129,130]
miR-155	Increased in β-cells in the elderly	IGF1-1/PI3K/AKT	It is differentially expressed because of CR	[109,110,131,164]
miR-16	Increased in β-cells in the elderly	IGF-1R	It is differentially expressed because of CR	[110,154,164]
miR-34a	Increased in aging. Circulating levels upregulated in age-related hearing loss and in age-related macular degeneration	SIRT1 mTOR	It is differentially expressed by CR	[113,114,152,153]
miR-124a	Increased in aging and in aged skin	AKT3/FOXA2 SIRT1	A low-protein diet reduced its expression in pancreatic islets of pregnant rats	[114,115,116,117,167]
miR-383	Increased in aging	IGF-1 and IGF-1R	It is downregulated by a high-fat diet in mice pancreatic islets	[114,121,122,123,124]
miR-130b	Decreased in aging	PI3K/AKT (through direct inhibition of PTEN)	Circulating levels increase after an intervention with polyunsaturated fatty acids	[114,118,121]
miR-181a	Decreased in aging	PI3K/AKT (through direct inhibition of PTEN) SIRT1	Its levels increase in skeletal muscle of monkeys under a CR regime	[114,119,148]
miR-223	Decreased in CD4+ T cells of old donors and reduced in postmenopausal women	IGF1R/FOXO3A PI3K/AKT mTOR	A high-protein regime decreases High-Density Lipoproteins-associated miR-223 levels	[132,133,159,168]
miR-17-92 cluster	Decreased with age	mTOR	The expression of this cluster is downregulated by CR in a mouse model of breast cancer	[134,135,165]
miR-145	Decreased in peripheral blood mononuclear cells with aging	IRS1/IRS2	Upregulated by a high-fat diet, although not by CR	[137,138,166]
miR-199	Decreased in aging rhesus monkeys	SIRT1	Unknown	[142,143]
miR-19b	Lower levels in octogenarians as compared with centenarians and young individuals	PGC1α	Circulating levels increase after an intervention with polyunsaturated fatty acids	[111,121]
miR-455	Decreased in old mice	AMPK	Upregulated in the liver of mice fed a high-fat diet	[145,146,147]
miR-451	Increased in aging primates	AMPK, mTORC1	Increased in primates under a CR regime	[148,149,164]
miR-142-3p	Decreased in postmenopausal women	IGF1R/FOXO3A PI3K/AKT mTOR	Upregulated by a high-fat diet	[130,159]
miR-182	Decreased in postmenopausal women	IGF1R/FOXO3A PI3K/AKT mTOR	Increased in the liver of mice fed a high-fat diet	[159,160]
miR-144	Increased in the skeletal muscle of old rhesus monkeys	PI3K/AKT (through direct inhibition of PTEN and IRS1) mTOR AMPK	Decreased in rats and monkeys under a CR regime	[149,155,156,157,158,163,164]
miR-221	Decreased by CR	PGC1α	It is downregulated by CR and polyunsaturated fatty acids	[121,149,151]

## Abbreviations

AD	Alzheimer’s disease
T2DM	Type 2 diabetes mellitus
CVD	Cardiovascular disease
CR	Caloric restriction
IF	Intermittent fasting
PA	Physical activity
RISC	RNA-induced silencing complex
PBMCs	Peripheral blood mononuclear cells
BAT	Brown adipose tissue
HFD	High-fat diet

## References

[1] Eurostat (2015). Yearbook.

[2] Willcox D.C., Scapagnini G., Willcox B.J. (2014). Healthy aging diets other than the mediterranean: A focus on the okinawan diet. Mech. Ageing Dev..

[3] Longo V.D., Antebi A., Bartke A., Barzilai N., Brown-Borg H.M., Caruso C., Curiel T.J., de Cabo R., Franceschi C., Gems D. (2015). Interventions to slow aging in humans: Are we ready?. Aging Cell.

[4] Lopez-Otin C., Blasco M.A., Partridge L., Serrano M., Kroemer G. (2013). The hallmarks of aging. Cell.

[5] Geller A.M., Zenick H. (2005). Aging and the environment: A research framework. Environ. Health Perspect..

[6] Most J., Tosti V., Redman L.M., Fontana L. (2016). Calorie restriction in humans: An update. Ageing Res. Rev..

[7] Zampetaki A., Willeit P., Drozdov I., Kiechl S., Mayr M. (2012). Profiling of circulating micrornas: From single biomarkers to re-wired networks. Cardiovasc. Res..

[8] Cui J., Zhou B., Ross S.A., Zempleni J. (2017). Nutrition, micrornas, and human health. Adv. Nutr..

[9] Ugalde A.P., Espanol Y., Lopez-Otin C. (2011). Micromanaging aging with mirnas: New messages from the nuclear envelope. Nucleus.

[10] Marin T., Gongol B., Chen Z., Woo B., Subramaniam S., Chien S., Shyy J.Y. (2013). Mechanosensitive micrornas-role in endothelial responses to shear stress and redox state. Free Radic. Biol. Med..

[11] Jung H.J., Suh Y. (2014). Regulation of IGF-1 signaling by micrornas. Front. Genet..

[12] Fontana L., Partridge L. (2015). Promoting health and longevity through diet: From model organisms to humans. Cell.

[13] Yu B.P., Masoro E.J., Murata I., Bertrand H.A., Lynd F.T. (1982). Life span study of SPF Fischer 344 male rats fed ad libitum or restricted diets: Longevity, growth, lean body mass and disease. J. Gerontol..

[14] Wohlgemuth S.E., Seo A.Y., Marzetti E., Lees H.A., Leeuwenburgh C. (2010). Skeletal muscle autophagy and apoptosis during aging: Effects of calorie restriction and life-long exercise. Exp. Gerontol..

[15] Donati A., Recchia G., Cavallini G., Bergamini E. (2008). Effect of aging and anti-aging caloric restriction on the endocrine regulation of rat liver autophagy. J. Geront. A Biol. Sci. Med. Sci..

[16] Anson R.M., Guo Z., de Cabo R., Iyun T., Rios M., Hagepanos A., Ingram D.K., Lane M.A., Mattson M.P. (2003). Intermittent fasting dissociates beneficial effects of dietary restriction on glucose metabolism and neuronal resistance to injury from calorie intake. Proc. Natl. Acad. Sci. USA.

[17] Varady K.A., Roohk D.J., Hellerstein M.K. (2007). Dose effects of modified alternate-day fasting regimens on in vivo cell proliferation and plasma insulin-like growth factor-1 in mice. J. Appl. Physiol..

[18] Varady K.A., Roohk D.J., Loe Y.C., McEvoy-Hein B.K., Hellerstein M.K. (2007). Effects of modified alternate-day fasting regimens on adipocyte size, triglyceride metabolism, and plasma adiponectin levels in mice. J. Lipid Res..

[19] Swindell W.R. (2012). Dietary restriction in rats and mice: A meta-analysis and review of the evidence for genotype-dependent effects on lifespan. Ageing Res. Rev..

[20] Kaeberlein T.L., Smith E.D., Tsuchiya M., Welton K.L., Thomas J.H., Fields S., Kennedy B.K., Kaeberlein M. (2006). Lifespan extension in *Caenorhabditis elegans* by complete removal of food. Aging Cell.

[21] Lee G.D., Wilson M.A., Zhu M., Wolkow C.A., de Cabo R., Ingram D.K., Zou S. (2006). Dietary deprivation extends lifespan in *Caenorhabditis elegans*. Aging Cell.

[22] Colman R.J., Beasley T.M., Kemnitz J.W., Johnson S.C., Weindruch R., Anderson R.M. (2014). Caloric restriction reduces age-related and all-cause mortality in rhesus monkeys. Nat. Commun..

[23] Mattison J.A., Roth G.S., Beasley T.M., Tilmont E.M., Handy A.M., Herbert R.L., Longo D.L., Allison D.B., Young J.E., Bryant M. (2012). Impact of caloric restriction on health and survival in rhesus monkeys from the NIA study. Nature.

[24] Maxmen A. (2012). Calorie restriction falters in the long run. Nature.

[25] Suzuki M., Wilcox B.J., Wilcox C.D. (2001). Implications from and for food cultures for cardiovascular disease: Longevity. Asia Pac. J. Clin. Nutr..

[26] Chatzianagnostou K., Del Turco S., Pingitore A., Sabatino L., Vassalle C. (2015). The Mediterranean Lifestyle as a Non-Pharmacological and Natural Antioxidant for Healthy Aging. Antioxidants.

[27] Estruch R., Ros E., Salas-Salvado J., Covas M.I., Corella D., Aros F., Gomez-Gracia E., Ruiz-Gutierrez V., Fiol M., Lapetra J. (2013). Primary prevention of cardiovascular disease with a mediterranean diet. N. Engl. J. Med..

[28] Garcia-Calzon S., Martinez-Gonzalez M.A., Razquin C., Aros F., Lapetra J., Martinez J.A., Zalba G., Marti A. (2016). Mediterranean diet and telomere length in high cardiovascular risk subjects from the PREDIMED-NAVARRA study. Clin. Nutr..

[29] Garcia-Calzon S., Gea A., Razquin C., Corella D., Lamuela-Raventos R.M., Martinez J.A., Martinez-Gonzalez M.A., Zalba G., Marti A. (2014). Longitudinal association of telomere length and obesity indices in an intervention study with a Mediterranean diet: The PREDIMED-NAVARRA trial. Int. J. Obes..

[30] Masana M.F., Koyanagi A., Haro J.M., Tyrovolas S. (2017). n-3 Fatty acids, mediterranean diet and cognitive function in normal aging: A systematic review. Exp. Gerontol..

[31] Nishihira J., Tokashiki T., Higashiuesato Y., Willcox D.C., Mattek N., Shinto L., Ohya Y., Dodge H.H. (2016). Associations between serum ω-3 fatty acid levels and cognitive functions among community-dwelling octogenarians in Okinawa, Japan: The KOCOA Study. J. Alzheimer Dis..

[32] Heilbronn L.K., de Jonge L., Frisard M.I., DeLany J.P., Larson-Meyer D.E., Rood J., Nguyen T., Martin C.K., Volaufova J., Most M.M. (2006). Effect of 6-month calorie restriction on biomarkers of longevity, metabolic adaptation, and oxidative stress in overweight individuals: A randomized controlled trial. JAMA.

[33] Ravussin E., Redman L.M., Rochon J., Das S.K., Fontana L., Kraus W.E., Romashkan S., Williamson D.A., Meydani S.N., Villareal D.T. (2015). A 2-year randomized controlled trial of human caloric restriction: Feasibility and effects on predictors of health span and longevity. J. Gerontol. A Biol. Sci. Med. Sci..

[34] Larson-Meyer D.E., Newcomer B.R., Heilbronn L.K., Volaufova J., Smith S.R., Alfonso A.J., Lefevre M., Rood J.C., Williamson D.A., Ravussin E. (2008). Effect of 6-month calorie restriction and exercise on serum and liver lipids and markers of liver function. Obesity.

[35] Lefevre M., Redman L.M., Heilbronn L.K., Smith J.V., Martin C.K., Rood J.C., Greenway F.L., Williamson D.A., Smith S.R., Ravussin E. (2009). Caloric restriction alone and with exercise improves CVD risk in healthy non-obese individuals. Atherosclerosis.

[36] Redman L.M., Veldhuis J.D., Rood J., Smith S.R., Williamson D., Ravussin E. (2010). The effect of caloric restriction interventions on growth hormone secretion in nonobese men and women. Aging Cell.

[37] Tam C.S., Frost E.A., Xie W., Rood J., Ravussin E., Redman L.M. (2014). No effect of caloric restriction on salivary cortisol levels in overweight men and women. Metabolism.

[38] Redman L.M., Heilbronn L.K., Martin C.K., de Jonge L., Williamson D.A., Delany J.P., Ravussin E. (2009). Metabolic and behavioral compensations in response to caloric restriction: Implications for the maintenance of weight loss. PLoS ONE.

[39] Williamson D.A., Martin C.K., Anton S.D., York-Crowe E., Han H., Redman L., Ravussin E. (2008). Is caloric restriction associated with development of eating-disorder symptoms? Results from the CALERIE trial. Health Psychol..

[40] Fontana L., Meyer T.E., Klein S., Holloszy J.O. (2004). Long-term calorie restriction is highly effective in reducing the risk for atherosclerosis in humans. Proc. Natl. Acad. Sci. USA.

[41] Fontana L., Klein S., Holloszy J.O. (2010). Effects of long-term calorie restriction and endurance exercise on glucose tolerance, insulin action, and adipokine production. Age.

[42] Soare A., Cangemi R., Omodei D., Holloszy J.O., Fontana L. (2011). Long-term calorie restriction, but not endurance exercise, lowers core body temperature in humans. Aging.

[43] Heilbronn L.K., Smith S.R., Martin C.K., Anton S.D., Ravussin E. (2005). Alternate-day fasting in nonobese subjects: Effects on body weight, body composition, and energy metabolism. Am. J. Clin. Nutr..

[44] Heilbronn L.K., Civitarese A.E., Bogacka I., Smith S.R., Hulver M., Ravussin E. (2005). Glucose tolerance and skeletal muscle gene expression in response to alternate day fasting. Obes. Res..

[45] Hoddy K.K., Bhutani S., Phillips S.A., Varady K.A. (2016). Effects of different degrees of insulin resistance on endothelial function in obese adults undergoing alternate day fasting. Nutr. Healthy Aging.

[46] Harder-Lauridsen N.M., Nielsen S.T., Mann S.P., Lyngbaek M.P., Benatti F.B., Langkilde A.R., Law I., Wedell-Neergaard A.S., Thomsen C., Moller K. (2017). The effect of alternate-day caloric restriction on the metabolic consequences of 8 days of bed rest in healthy lean men: A randomized trial. J. Appl. Physiol..

[47] Harvie M.N., Pegington M., Mattson M.P., Frystyk J., Dillon B., Evans G., Cuzick J., Jebb S.A., Martin B., Cutler R.G. (2011). The effects of intermittent or continuous energy restriction on weight loss and metabolic disease risk markers: A randomized trial in young overweight women. Int. J. Obes..

[48] Lettieri-Barbato D., Giovannetti E., Aquilano K. (2016). Effects of dietary restriction on adipose mass and biomarkers of healthy aging in human. Aging.

[49] Weiss E.P., Racette S.B., Villareal D.T., Fontana L., Steger-May K., Schechtman K.B., Klein S., Ehsani A.A., Holloszy J.O. (2007). Lower extremity muscle size and strength and aerobic capacity decrease with caloric restriction but not with exercise-induced weight loss. J. Appl. Physiol..

[50] Mirisola M.G., Taormina G., Fabrizio P., Wei M., Hu J., Longo V.D. (2014). Serine- and threonine/valine-dependent activation of PDK and Tor orthologs converge on Sch9 to promote aging. PLoS Genet..

[51] Mair W., Piper M.D., Partridge L. (2005). Calories do not explain extension of life span by dietary restriction in Drosophila. PLoS Biol..

[52] Segall P.E., Timiras P.S. (1976). Patho-physiologic findings after chronic tryptophan deficiency in rats: A model for delayed growth and aging. Mech. Ageing Dev..

[53] Ooka H., Segall P.E., Timiras P.S. (1988). Histology and survival in age-delayed low-tryptophan-fed rats. Mech. Ageing Dev..

[54] Orentreich N., Matias J.R., DeFelice A., Zimmerman J.A. (1993). Low methionine ingestion by rats extends life span. J. Nutr..

[55] Miller R.A., Buehner G., Chang Y., Harper J.M., Sigler R., Smith-Wheelock M. (2005). Methionine-deficient diet extends mouse lifespan, slows immune and lens aging, alters glucose, T4, IGF-I and insulin levels, and increases hepatocyte MIF levels and stress resistance. Aging Cell.

[56] Gallinetti J., Harputlugil E., Mitchell J.R. (2013). Amino acid sensing in dietary-restriction-mediated longevity: Roles of signal-transducing kinases GCN2 and TOR. Biochem. J..

[57] Johnson S.C., Rabinovitch P.S., Kaeberlein M. (2013). mTOR is a key modulator of ageing and age-related disease. Nature.

[58] Altintas O., Park S., Lee S.J. (2016). The role of insulin/IGF-1 signaling in the longevity of model invertebrates, *C. elegans* and *D. melanogaster*. BMB Rep..

[59] Martins R., Lithgow G.J., Link W. (2016). Long live FOXO: Unraveling the role of FOXO proteins in aging and longevity. Aging Cell.

[60] Harrison D.E., Strong R., Sharp Z.D., Nelson J.F., Astle C.M., Flurkey K., Nadon N.L., Wilkinson J.E., Frenkel K., Carter C.S. (2009). Rapamycin fed late in life extends lifespan in genetically heterogeneous mice. Nature.

[61] Miller R.A., Harrison D.E., Astle C.M., Fernandez E., Flurkey K., Han M., Javors M.A., Li X., Nadon N.L., Nelson J.F. (2014). Rapamycin-mediated lifespan increase in mice is dose and sex dependent and metabolically distinct from dietary restriction. Aging Cell.

[62] Tullet J.M., Hertweck M., An J.H., Baker J., Hwang J.Y., Liu S., Oliveira R.P., Baumeister R., Blackwell T.K. (2008). Direct inhibition of the longevity-promoting factor SKN-1 by insulin-like signaling in *C. elegans*. Cell.

[63] Uno M., Honjoh S., Matsuda M., Hoshikawa H., Kishimoto S., Yamamoto T., Ebisuya M., Matsumoto K., Nishida E. (2013). A fasting-responsive signaling pathway that extends life span in *C. elegans*. Cell Rep..

[64] Giannakou M.E., Goss M., Partridge L. (2008). Role of dFOXO in lifespan extension by dietary restriction in Drosophila melanogaster: Not required, but its activity modulates the response. Aging Cell.

[65] Shimokawa I., Komatsu T., Hayashi N., Kim S.E., Kawata T., Park S., Hayashi H., Yamaza H., Chiba T., Mori R. (2015). The life-extending effect of dietary restriction requires Foxo3 in mice. Aging Cell.

[66] Warr M.R., Binnewies M., Flach J., Reynaud D., Garg T., Malhotra R., Debnath J., Passegue E. (2013). FOXO3A directs a protective autophagy program in haematopoietic stem cells. Nature.

[67] Bao J.M., Song X.L., Hong Y.Q., Zhu H.L., Li C., Zhang T., Chen W., Zhao S.C., Chen Q. (2014). Association between *FOXO3A* gene polymorphisms and human longevity: A meta-analysis. Asian J. Androl..

[68] Willcox B.J., Tranah G.J., Chen R., Morris B.J., Masaki K.H., He Q., Willcox D.C., Allsopp R.C., Moisyadi S., Poon L.W. (2016). The *FoxO3* gene and cause-specific mortality. Aging Cell.

[69] Mercken E.M., Crosby S.D., Lamming D.W., JeBailey L., Krzysik-Walker S., Villareal D.T., Capri M., Franceschi C., Zhang Y., Becker K. (2013). Calorie restriction in humans inhibits the PI3K/AKT pathway and induces a younger transcription profile. Aging Cell.

[70] Cheng C.W., Adams G.B., Perin L., Wei M., Zhou X., Lam B.S., da Sacco S., Mirisola M., Quinn D.I., Dorff T.B. (2014). Prolonged fasting reduces IGF-1/PKA to promote hematopoietic-stem-cell-based regeneration and reverse immunosuppression. Cell Stem Cell.

[71] Kenyon C.J. (2010). The genetics of ageing. Nature.

[72] Jia K., Chen D., Riddle D.L. (2004). The TOR pathway interacts with the insulin signaling pathway to regulate *C. elegans* larval development, metabolism and life span. Development.

[73] Honjoh S., Yamamoto T., Uno M., Nishida E. (2009). Signalling through RHEB-1 mediates intermittent fasting-induced longevity in *C. elegans*. Nature.

[74] Fontana L., Adelaiye R.M., Rastelli A.L., Miles K.M., Ciamporcero E., Longo V.D., Nguyen H., Vessella R., Pili R. (2013). Dietary protein restriction inhibits tumor growth in human xenograft models. Oncotarget.

[75] Solon-Biet S.M., McMahon A.C., Ballard J.W., Ruohonen K., Wu L.E., Cogger V.C., Warren A., Huang X., Pichaud N., Melvin R.G. (2014). The ratio of macronutrients, not caloric intake, dictates cardiometabolic health, aging, and longevity in ad libitum-fed mice. Cell Metab..

[76] Fontana L., Weiss E.P., Villareal D.T., Klein S., Holloszy J.O. (2008). Long-term effects of calorie or protein restriction on serum IGF-1 and IGFBP-3 concentration in humans. Aging Cell.

[77] Suh Y., Atzmon G., Cho M.O., Hwang D., Liu B., Leahy D.J., Barzilai N., Cohen P. (2008). Functionally significant insulin-like growth factor I receptor mutations in centenarians. Proc. Natl. Acad. Sci. USA.

[78] Milman S., Atzmon G., Huffman D.M., Wan J., Crandall J.P., Cohen P., Barzilai N. (2014). Low insulin-like growth factor-1 level predicts survival in humans with exceptional longevity. Aging Cell.

[79] Allard J.S., Heilbronn L.K., Smith C., Hunt N.D., Ingram D.K., Ravussin E., de Cabo R. (2008). In vitro cellular adaptations of indicators of longevity in response to treatment with serum collected from humans on calorie restricted diets. PLoS ONE.

[80] Canto C., Auwerx J. (2012). Targeting sirtuin 1 to improve metabolism: All you need is NAD^+^?. Pharmacol. Rev..

[81] Lin S.J., Defossez P.A., Guarente L. (2000). Requirement of nad and sir2 for life-span extension by calorie restriction in saccharomyces cerevisiae. Science.

[82] Scheibye-Knudsen M., Mitchell S.J., Fang E.F., Iyama T., Ward T., Wang J., Dunn C.A., Singh N., Veith S., Hasan-Olive M.M. (2014). A high-fat diet and NAD^+^ activate Sirt1 to rescue premature aging in cockayne syndrome. Cell Metab..

[83] Satoh A., Brace C.S., Ben-Josef G., West T., Wozniak D.F., Holtzman D.M., Herzog E.D., Imai S. (2010). SIRT1 promotes the central adaptive response to diet restriction through activation of the dorsomedial and lateral nuclei of the hypothalamus. J. Neurosci..

[84] Satoh A., Brace C.S., Rensing N., Cliften P., Wozniak D.F., Herzog E.D., Yamada K.A., Imai S. (2013). Sirt1 extends life span and delays aging in mice through the regulation of Nk2 homeobox 1 in the DMH and LH. Cell Metab..

[85] Mouchiroud L., Houtkooper R.H., Auwerx J. (2013). NAD^+^ metabolism: A therapeutic target for age-related metabolic disease. Crit. Rev. Biochem. Mol. Biol..

[86] Howitz K.T., Bitterman K.J., Cohen H.Y., Lamming D.W., Lavu S., Wood J.G., Zipkin R.E., Chung P., Kisielewski A., Zhang L.L. (2003). Small molecule activators of sirtuins extend Saccharomyces cerevisiae lifespan. Nature.

[87] Novelle M.G., Ali A., Dieguez C., Bernier M., de Cabo R. (2016). Metformin: A hopeful promise in aging research. Cold Spring Harb. Perspect. Med..

[88] Wu J.W., Boudreau D.M., Park Y., Simonds N.I., Freedman A.N. (2014). Commonly used diabetes and cardiovascular medications and cancer recurrence and cancer-specific mortality: A review of the literature. Expert Opin. Drug Saf..

[89] Ng T.P., Feng L., Yap K.B., Lee T.S., Tan C.H., Winblad B. (2014). Long-term metformin usage and cognitive function among older adults with diabetes. J. Alzheimer Dis..

[90] Zhang C., Li S., Yang L., Huang P., Li W., Wang S., Zhao G., Zhang M., Pang X., Yan Z. (2013). Structural modulation of gut microbiota in life-long calorie-restricted mice. Nat. Commun..

[91] Breen D.M., Rasmussen B.A., Cote C.D., Jackson V.M., Lam T.K. (2013). Nutrient-sensing mechanisms in the gut as therapeutic targets for diabetes. Diabetes.

[92] Efeyan A., Comb W.C., Sabatini D.M. (2015). Nutrient-sensing mechanisms and pathways. Nature.

[93] Vrieze A., Van Nood E., Holleman F., Salojarvi J., Kootte R.S., Bartelsman J.F., Dallinga-Thie G.M., Ackermans M.T., Serlie M.J., Oozeer R. (2012). Transfer of intestinal microbiota from lean donors increases insulin sensitivity in individuals with metabolic syndrome. Gastroenterology.

[94] Garcia-Segura L., Perez-Andrade M., Miranda-Rios J. (2013). The emerging role of microRNAs in the regulation of gene expression by nutrients. J. Nutrigenet. Nutrigenom..

[95] Choi S.W., Claycombe K.J., Martinez J.A., Friso S., Schalinske K.L. (2013). Nutritional epigenomics: A portal to disease prevention. Adv. Nutr..

[96] Catalanotto C., Cogoni C., Zardo G. (2016). MicroRNA in control of gene expression: An overview of nuclear functions. Int. J. Mol. Sci..

[97] Winter J., Jung S., Keller S., Gregory R.I., Diederichs S. (2009). Many roads to maturity: MicroRNA biogenesis pathways and their regulation. Nat. Cell Biol..

[98] Suzuki H.I., Yamagata K., Sugimoto K., Iwamoto T., Kato S., Miyazono K. (2009). Modulation of microRNA processing by p53. Nature.

[99] Davalos A., Goedeke L., Smibert P., Ramirez C.M., Warrier N.P., Andreo U., Cirera-Salinas D., Rayner K., Suresh U., Pastor-Pareja J.C. (2011). miR-33a/b contribute to the regulation of fatty acid metabolism and insulin signaling. Proc. Natl. Acad. Sci. USA.

[100] Rayner K.J., Suarez Y., Davalos A., Parathath S., Fitzgerald M.L., Tamehiro N., Fisher E.A., Moore K.J., Fernandez-Hernando C. (2010). MiR-33 contributes to the regulation of cholesterol homeostasis. Science.

[101] Saeidimehr S., Ebrahimi A., Saki N., Goodarzi P., Rahim F. (2016). MicroRNA-based linkage between aging and cancer: From epigenetics view point. Cell J..

[102] Frost R.J., Olson E.N. (2011). Control of glucose homeostasis and insulin sensitivity by the Let-7 family of microRNAs. Proc. Natl. Acad. Sci. USA.

[103] Zhu H., Shyh-Chang N., Segrè A.V., Shinoda G., Shah S.P., Einhorn W.S., Takeuchi A., Engreitz J.M., Hagan J.P., Kharas M.G. (2011). The Lin28/let-7 axis regulates glucose metabolism. Cell.

[104] Pullen T.J., da Silva Xavier G., Kelsey G., Rutter G.A. (2011). miR-29a and miR-29b contribute to pancreatic β-cell-specific silencing of monocarboxylate transporter 1 (Mct1). Mol. Cell. Biol..

[105] Trajkovski M., Hausser J., Soutschek J., Bhat B., Akin A., Zavolan M., Heim M.H., Stoffel M. (2011). MicroRNAs 103 and 107 regulate insulin sensitivity. Nature.

[106] Li Y.Q., Zhang M.F., Wen H.Y., Hu C.L., Liu R., Wei H.Y., Ai C.M., Wang G., Liao X.X., Li X. (2013). Comparing the diagnostic values of circulating microRNAs and cardiac troponin T in patients with acute myocardial infarction. Clinics.

[107] Goren Y., Kushnir M., Zafrir B., Tabak S., Lewis B.S., Amir O. (2012). Serum levels of microRNAs in patients with heart failure. Eur. J. Heart Fail..

[108] Tijsen A.J., Creemers E.E., Moerland P.D., de Windt L.J., van der Wal A.C., Kok W.E., Pinto Y.M. (2010). Mir423-5p as a circulating biomarker for heart failure. Circ. Res..

[109] Ma X., Becker Buscaglia L.E., Barker J.R., Li Y. (2011). MicroRNAs in NF-κB signaling. J. Mol. Cell Biol..

[110] Frasca D., Diaz A., Romero M., Ferracci F., Blomberg B.B. (2015). MicroRNAs miR-155 and miR-16 decrease AID and E47 in B cells from elderly individuals. J. Immunol..

[111] Serna E., Gambini J., Borras C., Abdelaziz K.M., Mohammed K., Belenguer A., Sanchis P., Avellana J.A., Rodriguez-Mañas L., Viña J. (2012). Centenarians, but not octogenarians, up-regulate the expression of microRNAs. Sci. Rep..

[112] Olivieri F., Capri M., Bonafè M., Morsiani C., Jung H.J., Spazzafumo L., Viña J., Suh Y. (2016). Circulating miRNAs and miRNA shuttles as biomarkers: Perspective trajectories of healthy and unhealthy aging. Mech. Ageing Dev..

[113] Pang J., Xiong H., Yang H., Ou Y., Xu Y., Huang Q., Lai L., Chen S., Zhang Z., Cai Y. (2016). Circulating miR-34a levels correlate with age-related hearing loss in mice and humans. Exp. Gerontol..

[114] Tugay K., Guay C., Marques A.C., Allagnat F., Locke J.M., Harries L.W., Rutter G.A., Regazzi R. (2016). Role of microRNAs in the age-associated decline of pancreatic β cell function in rat islets. Diabetologia.

[115] Sebastiani G., Po A., Miele E., Ventriglia G., Ceccarelli E., Bugliani M., Marselli L., Marchetti P., Gulino A., Ferretti E. (2015). MicroRNA-124a is hyperexpressed in type 2 diabetic human pancreatic islets and negatively regulates insulin secretion. Acta Diabetol..

[116] Gong X., Wang H., Ye Y., Shu Y., Deng Y., He X., Lu G., Zhang S. (2016). miR-124 regulates cell apoptosis and autophagy in dopaminergic neurons and protects them by regulating AMPK/mTOR pathway in Parkinson's disease. Am. J. Transl. Res..

[117] Harada M., Jinnin M., Wang Z., Hirano A., Tomizawa Y., Kira T., Igata T., Masuguchi S., Fukushima S., Ihn H. (2017). The expression of miR-124 increases in aged skin to cause cell senescence and it decreases in squamous cell carcinoma. Biosci. Trends.

[118] Miao Y., Zheng W., Li N., Su Z., Zhao L., Zhou H., Jia L. (2017). MicroRNA-130b targets *PTEN* to mediate drug resistance and proliferation of breast cancer cells via the PI3K/Akt signaling pathway. Sci. Rep..

[119] Zhang X., Li X., Tan F., Yu N., Pei H. (2017). STAT1 Inhibits miR-181a expression to suppress colorectal cancer cell proliferation through PTEN/Akt. J. Cell. Biochem..

[120] Zhou B., Li C., Qi W., Zhang Y., Zhang F., Wu J.X., Hu Y.N., Wu D.M., Liu Y., Yan T.T. (2012). Downregulation of miR-181a upregulates sirtuin-1 (*SIRT1*) and improves hepatic insulin sensitivity. Diabetologia.

[121] Ortega F.J., Cardona-Alvarado M.I., Mercader J.M., Moreno-Navarrete J.M., Moreno M., Sabater M., Fuentes-Batllevell N., Ramirez-Chavez E., Ricart W., Molina-Torres J. (2015). Circulating profiling reveals the effect of a polyunsaturated fatty acid-enriched diet on common microRNAs. J. Nutr. Biochem..

[122] Berber P., Grassmann F., Kiel C., Weber B.H. (2017). An eye on age-related macular degeneration: The role of microRNAs in disease pathology. Mol. Diagn. Ther..

[123] Chakraborty C., Doss C.G., Bandyopadhyay S., Agoramoorthy G. (2014). Influence of miRNA in insulin signaling pathway and insulin resistance: Micro-molecules with a major role in type-2 diabetes. WIREs RNA.

[124] Nesca V., Guay C., Jacovetti C., Menoud V., Peyot M.L., Laybutt D.R., Prentki M., Regazzi R. (2013). Identification of particular groups of microRNAs that positively or negatively impact on β cell function in obese models of type 2 diabetes. Diabetologia.

[125] Xu F., Pang L., Cai X., Liu X., Yuan S., Fan X., Jiang B., Zhang X., Dou Y., Gorospe M. (2014). let-7-repressesed *SHC* translation delays replicative senescence. Aging Cell.

[126] Hyun S., Lee J.H., Jin H., Nam J., Namkoong B., Lee G., Chung J., Kim V.N. (2009). Conserved MicroRNA miR-8/miR-200 and its target *USH/FOG2* control growth by regulating PI3K. Cell.

[127] Ma M., Wang X., Chen X., Cai R., Chen F., Dong W., Yang G., Pang W. (2017). MicroRNA-432 targeting E2F3 and P55PIK inhibits myogenesis through PI3K/AKT/mTOR signaling pathway. RNA Biol..

[128] Han C., Shen J.K., Hornicek F.J., Kan Q., Duan Z. (2017). Regulation of microRNA-1 (miR-1) expression in human cancer. Biochim. Biophys. Acta.

[129] Mariño G., Ugalde A.P., Fernández A.F., Osorio F.G., Fueyo A., Freije J.M., López-Otín C. (2010). Insulin-like growth factor 1 treatment extends longevity in a mouse model of human premature aging by restoring somatotroph axis function. Proc. Natl. Acad. Sci. USA.

[130] Chartoumpekis D.V., Zaravinos A., Ziros P.G., Iskrenova R.P., Psyrogiannis A.I., Kyriazopoulou V.E., Habeos I.G. (2012). Differential expression of microRNAs in adipose tissue after long-term high-fat diet-induced obesity in mice. PLoS ONE.

[131] Lin X., Qin Y., Jia J., Lin T., Chen L., Zeng H., Han Y., Wu L., Huang S., Wang M. (2016). MiR-155 enhances insulin sensitivity by coordinated regulation of multiple genes in mice. PLoS Genet..

[132] Gao H., Deng H., Xu H., Yang Q., Zhou Y., Zhang J., Zhao D., Liu F. (2016). MicroRNA-223 promotes mast cell apoptosis by targeting the insulin-like growth factor 1 receptor. Exp. Ther. Med..

[133] Teteloshvili N., Kluiver J., van der Geest K.S., van der Lei R.J., Jellema P., Pawelec G., Brouwer E., Kroesen B.J., Boots A.M., van den Berg A. (2015). Age-associated differences in miRNA signatures are restricted to CD45RO negative T cells and are associated with changes in the cellular composition, activation and cellular ageing. PLoS ONE.

[134] Xie R., Lin X., Du T., Xu K., Shen H., Wei F., Hao W., Lin T., Qin Y., Wang H. (2016). Targeted Disruption of miR-17-92 impairs mouse spermatogenesis by activating mTOR signaling pathway. Medicine.

[135] Grillari J., Hackl M., Grillari-Voglauer R. (2010). miR-17–92 cluster: Ups and downs in cancer and aging. Biogerontology.

[136] Meyer S.U., Thirion C., Polesskaya A., Bauersachs S., Kaiser S., Krause S., Pfaffl M.W. (2015). TNF-α and IGF1 modify the microRNA signature in skeletal muscle cell differentiation. Cell Commun. Signal..

[137] Law P.T., Ching A.K., Chan A.W., Wong Q.W., Wong C.K., To K.F., Wong N. (2012). MiR-145 modulates multiple components of the insulin-like growth factor pathway in hepatocellular carcinoma. Carcinogenesis.

[138] Budzinska M., Owczarz M., Pawlik-Pachucka E., Roszkowska-Gancarz M., Slusarczyk P., Puzianowska-Kuznicka M. (2016). MiR-96, miR-145 and miR-9 expression increases, and IGF-1R and FOXO1 expression decreases in peripheral blood mononuclear cells of aging humans. BMC Geriatr..

[139] Xie H., Lei N., Gong A.Y., Chen X.M., Hu G. (2014). Cryptosporidium parvum induces SIRT1 expression in host epithelial cells through downregulating let-7i. Hum. Immunol..

[140] Menghini R., Casagrande V., Cardellini M., Martelli E., Terrinoni A., Amati F., Vasa-Nicotera M., Ippoliti A., Novelli G., Melino G. (2009). Microrna 217 modulates endothelial cell senescence via silent information regulator 1. Circulation.

[141] Kurylowicz A., Owczarz M., Polosak J., Jonas M.I., Lisik W., Jonas M., Chmura A., Puzianowska-Kuznicka M. (2016). *SIRT1* and *SIRT7* expression in adipose tissues of obese and normal-weight individuals is regulated by microRNAs but not by methylation status. Int. J. Obes..

[142] Yamac A.H., Kucukbuzcu S., Ozansoy M., Gok O., Oz K., Erturk M., Yilmaz E., Ersoy B., Zeybek R., Goktekin O. (2016). Altered expression of micro-RNA 199a and increased levels of cardiac SIRT1 protein are associated with the occurrence of atrial fibrillation after coronary artery bypass graft surgery. Cardiovasc. Pathol..

[143] Yu J.M., Wu X., Gimble J.M., Guan X., Freitas M.A., Bunnell B.A. (2011). Age-related changes in mesenchymal stem cells derived from rhesus macaque bone marrow. Aging Cell.

[144] Xue Y., Wei Z., Ding H., Wang Q., Zhou Z., Zheng S., Zhang Y., Hou D., Liu Y., Zen K. (2015). MicroRNA-19b/221/222 induces endothelial cell dysfunction via suppression of *PGC-1α* in the progression of atherosclerosis. Atherosclerosis.

[145] Zhang H., Guan M., Townsend K.L., Huang T.L., An D., Yan X., Xue R., Schulz T.J., Winnay J., Mori M. (2015). MicroRNA-455 regulates brown adipogenesis via a novel HIF1an-AMPK-PGC1α signaling network. EMBO Rep..

[146] Nidadavolu L.S., Niedernhofer L.J., Khan S.A. (2013). Identification of microRNAs dysregulated in cellular senescence driven by endogenous genotoxic stress. Aging.

[147] Yang W.M., Min K.H., Lee W. (2016). MicroRNA expression analysis in the liver of high fat diet-induced obese mice. Data Brief.

[148] Chen M.B., Wei M.X., Han J.Y., Wu X.Y., Li C., Wang J., Shen W., Lu P.H. (2014). MicroRNA-451 regulates AMPK/mTORC1 signaling and fascin1 expression in HT-29 colorectal cancer. Cell Signal..

[149] Mercken E.M., Majounie E., Ding J., Guo R., Kim J., Bernier M., Mattison J., Cookson M.R., Gorospe M., de Cabo R. (2013). Age-associated miRNA alterations in skeletal muscle from rhesus monkeys reversed by caloric restriction. Aging.

[150] Liu P., Hu Y., Ma L., Du M., Xia L., Hu Z. (2015). miR-425 inhibits melanoma metastasis through repression of PI3K-Akt pathway by targeting IGF-1. Biomed. Pharmacother..

[151] Ihle M.A., Trautmann M., Kuenstlinger H., Huss S., Heydt C., Fassunke J., Wardelmann E., Bauer S., Schildhaus H.U., Buettner R. (2015). miRNA-221 and miRNA-222 induce apoptosis via the KIT/AKT signalling pathway in gastrointestinal stromal tumours. Mol. Oncol..

[152] Iekushi K., Seeger F., Assmus B., Zeiher A.M., Dimmeler S. (2012). Regulation of cardiac microRNAs by bone marrow mononuclear cell therapy in myocardial infarction. Circulation.

[153] Kou X., Liu X., Chen X., Li J., Yang X., Fan J., Yang Y., Chen N. (2016). Ampelopsin attenuates brain aging of D-gal-induced rats through miR-34a-mediated SIRT1/mTOR signal pathway. Oncotarget.

[154] Chen L., Wang Q., Wang G.D., Wang H.S., Huang Y., Liu X.M., Cai X.H. (2013). miR-16 inhibits cell proliferation by targeting *IGF1R* and theRraf1-MEK1/2-ERK1/2 pathway in osteosarcoma. FEBS Lett..

[155] Zhang L.Y., Ho-Fun L.V., Wong A.M., Kwong D.L., Zhu Y.H., Dong S.S., Kong K.L., Chen J., Tsao S.W., Guan X.Y. (2013). MicroRNA-144 promotes cell proliferation, migration and invasion in nasopharyngeal carcinoma through repression of *PTEN*. Carcinogenesis.

[156] Wu X., Cui C.L., Chen W.L., Fu Z.Y., Cui X.Y., Gong X. (2016). miR-144 suppresses the growth and metastasis of laryngeal squamous cell carcinoma by targeting *IRS1*. Am. J. Transl. Res..

[157] Xiang C., Cui S.P., Ke Y. (2016). MiR-144 inhibits cell proliferation of renal cell carcinoma by targeting MTOR. J. Huazhong Univ. Sci. Technol. Med. Sci..

[158] Turczynska K.M., Bhattachariya A., Sall J., Goransson O., Sward K., Hellstrand P., Albinsson S. (2013). Stretch-sensitive down-regulation of the miR-144/451 cluster in vascular smooth muscle and its role in AMP-activated protein kinase signaling. PLoS ONE.

[159] Olivieri F., Ahtiainen M., Lazzarini R., Pöllänen E., Capri M., Lorenzi M., Fulgenzi G., Albertini M.C., Salvioli S., Alen M.J. (2014). Hormone replacement therapy enhances IGF-1 signaling in skeletal muscle by diminishing miR-182 and miR-223 expressions: A study on postmenopausal monozygotic twin pairs. Aging Cell.

[160] Tessitore A., Cicciarelli G., Del Vecchio F., Gaggiano A., Verzella D., Fischietti M., Mastroiaco V., Vetuschi A., Sferra R., Barnabei R. (2016). MicroRNA expression analysis in high fat diet-induced NAFLD-NASH-HCC progression: Study on C57BL/6J mice. BMC Cancer.

[161] Liu Z.Q., Fu W.Q., Zhao S., Zhao X. (2016). Regulation of insulin-like growth factor 1 receptor signaling by microRNA-4458 in the development of lumbar disc degeneration. Am. J. Transl. Res..

[162] Li X., Sun X., Wu J., Li Z. (2016). MicroRNA-613 suppresses proliferation, migration and invasion of osteosarcoma by targeting c-MET. Am. J. Cancer Res..

[163] Csiszar A., Gautam T., Sosnowska D., Tarantini S., Banki E., Tucsek Z., Toth P., Losonczy G., Koller A., Reglodi D. (2014). Caloric restriction confers persistent anti-oxidative, pro-angiogenic, and anti-inflammatory effects and promotes anti-aging miRNA expression profile in cerebromicrovascular endothelial cells of aged rats. Am. J. Physiol. Heart Circ. Physiol..

[164] Olivo-Marston S.E., Hursting S.D., Perkins S.N., Schetter A., Khan M., Croce C., Harris C.C., Lavigne J. (2014). Effects of calorie restriction and diet-induced obesity on murine colon carcinogenesis, growth and inflammatory factors, and microRNA expression. PLoS ONE.

[165] Jin L., Lim M., Zhao S., Sano Y., Simone B.A., Savage J.E., Wickstrom E., Camphausen K., Pestell R.G., Simone N.L. (2014). The metastatic potential of triple-negative breast cancer is decreased via caloric restriction-mediated reduction of the miR-17~92 cluster. Breast Cancer Res. Treat..

[166] Sangiao-Alvarellos S., Pena-Bello L., Manfredi-Lozano M., Tena-Sempere M., Cordido F. (2014). Perturbation of hypothalamic microRNA expression patterns in male rats after metabolic distress: Impact of obesity and conditions of negative energy balance. Endocrinology.

[167] De Siqueira K.C., de Lima F.M., Lima F.S., Taki M.S., da Cunha C.F., de Lima Reis S.R., Camargo R.L., Batista T.M., Vanzela E.C., Nardelli T.R. (2017). miR-124a expression contributes to the monophasic pattern of insulin secretion in islets from pregnant rats submitted to a low-protein diet. Eur. J. Nutr..

[168] Tabet F., Cuesta Torres L.F., Ong K.L., Shrestha S., Choteau S.A., Barter P.J., Clifton P., Rye K.A. (2016). High-Density Lipoprotein-Associated miR-223 is Altered after Diet-Induced Weight loss in Overweight and Obese Males. PLoS ONE.

